# CANCER CARE IN ELDERLY: CHALLENGES AND REQUIREMENTS OF INFORMAL CAREGIVERS

**DOI:** 10.1093/geroni/igac059.2240

**Published:** 2022-12-20

**Authors:** Esha Chakravarty, Indrani Chakravarty

**Affiliations:** Calcutta Metropolitan Institute of Gerontology, London, London, United Kingdom; Calcutta Metropolitan Institute of Gerontology, Kolkata, West Bengal, India

## Abstract

The affect of Covid -19 and changes in healthcare has shifted most of cancer care of elderly to home settings. Calcutta Metropolitan Institute of Gerontology (CMIG), Regional Resource and Training Centre in Ageing studied the daily tasks, requirements and emotional burden of caregivers in West Bengal, India. Caregivers of lung cancer, liver cancer and colorectal cancer patients (n=500) completed questionnaires provided by CMIG. The mean age of cancer patients was 72.5 years. CMIG found that 47% of the caregivers were the spouses of elderly patients, 33% were their adult children and 20% were other family members. Forty six percent of the caregivers were found to care for patients with metastatic disease, 33% cared for co morbidities, 63% cared for those undergoing chemotherapy or radiotherapy. Ninety eight percent of the caregivers provided assistance with Activities of Daily Living, 75% in administering medicine, 47% helped in pain management, 39% helped in hospital visits or deciding to call doctor. Twenty one percent of the caregivers reported poor health and 16% were not confident of the quality of care they provided. Sixty six percent reported suffering from stress and perceived cancer as a family disease, 45% thought they lacked social support. Of the adult children who provided care, 78% resported they needed more training to provide quality cancer care and transitioning to formal care would improve results.This study will help influence State policies for allocation of resources for training for cancer caregivers, build support groups for stress management and create cancer care helplines.

